# Supplementary material for: Analytic Pearson residuals for normalization of single-cell RNA-seq UMI data
Source: Genome Biol. 2021 Sep 6;22:258. doi: 10.1186/s13059-021-02451-7 (PMC8419999; doi:10.1186/s13059-021-02451-7)
Supplement: Supplementary file 2 — Additional file 2 Review history. [file 13059_2021_2451_MOESM2_ESM.docx]

Review History

**First round of review**

**Reviewer 1**

**Are you able to assess all statistics in the manuscript, including the appropriateness of statistical tests used?**

Yes, and I have assessed the statistics in my report.

**Comments to author:**

Referee report for GBIO-D-20-01910

Analytic Pearson residuals for normalization of single-cell RNA-seq UMI data

Lause et al discuss some of the challenges arising in the normalisation of UMI-based single cell RNA sequencing (scRNAseq). The authors focus on specific issues arising from the model introduced by Hafemeister and Satija [1]. In particular, the authors argue that the model presented in [1] is over-specified and highlight challenges in the estimation of gene-specific over-dispersion parameters. Lause et al propose a simplification of the model in [1], which enables a close form solution for the associated Pearson residuals. This simplified model is shown to match a special case of the GLM-PCA method presented by Townes et al [2]. Finally, the authors compare the performance between the Pearson residuals mentioned above and an existing approach for normalisation and transformation of scRNAseq data prior to downstream analysis such as dimensionality reduction and clustering.

The authors raise an interesting discussion about the analysis of UMI-based scRNAseq data. However, I believe that some aspects of the analysis need to be improved before the article can be considered for publication. Moreover, some relevant literature is missing and direct links with other existing bulk and scRNAseq analysis tools is not provided (e.g. for the rank-one GLM-PCA model).Detailed comments are provided below.

Major comments

1.	Equation (3). This formulation matches what is known as global-scaling normalisation, where cell-specific scaling factors are used to capture overall differences across cells (e.g. sequencing depth or mRNA content). Such strategy is widely used in the context of bulk (e.g. [3]) and scRNAseq (e.g. [4]) analysis tools. As such, the authors should provide links to the relevant literature. Please note that whilst [1] and Lause et al use sequencing depth as cell-specific offsets ($n_c$), their use has been shown to be problematic and suitable alternatives (e.g. [4]) have been proposed.

2.	Page 3, line 25 (intercepts that showed zero variance). This statement is not accurate and needs to be clarified. Whilst the authors do observe that the intercept estimates are a deterministic function of the log-expression, this does imply that the associated estimator has “zero variance”. In fact, these estimates are subject to the same source of frequentist variance (e.g. as reflected by standard errors) as in maximum likelihood estimates. I suggest to rephrase as “intercept estimates were a deterministic function of log-expression (Figure 1e) as described in equation (4).

3.	Equation (8). The proposed “rank-one GLM-PCA” approach matches existing count-based models used for bulk (e.g. [3]) and scRNAseq (e.g. [5]) data. In fact, the main difference relates to the strategy used to obtain parameter estimates (e.g. different choices for cell-specific scaling factors, regularisation of over-dispersion parameters). It is important to describe these links as the proposed model is not novel in itself, the main novelty relates to the explicit derivation of Pearson residuals (which is not done as a default option in the existing implementation of the existent models).

4.	Page 4. The authors argue that the procedure in [1] leads to biased over-dispersion estimates. This observation is supported by empirical results obtained from synthetic data and by highlighting convergence issues for the underlying estimation procedure.

a.	Maximum likelihood estimation for NB over-dispersion parameters is indeed problematic for lowly expressed genes and small sample sizes. This fact has been documented in the context of bulk [3] and scRNAseq [5] analysis, and suitable solutions have been proposed. Therefore, whilst the proposal of using a constant over-dispersion value across all genes may be a pragmatic solution to avoid the effect of this bias in the context of Pearson residuals, this is not necessarily required and better over-dispersion estimates could be used.

b.	The evidence provided using synthetic data and negative control datasets is not sufficient to support the use of a constant over-dispersion value across all genes. Whilst this may not have major consequences when deriving Pearson residuals, this does imply that a model with constant over-dispersion provides a better fit to real datasets in which underlying biological variability is present. To assess this, the strategy implemented by [6] could be used (the latter compares summary statistics derived from the raw data to those obtained from synthetic datasets generated by a range of competing models).

c.	For the PBMC dataset, the reported estimation issues will primarily affect lowly

expressed genes whose expression was only captured in a handful of cells. The current inclusion criteria applied by the authors is very liberal (16809 genes that were detected in at least five cells – as in [1]) and a more stringent filter will likely attenuate the issue.

5.	Figure 2a. A critical limitation for the chosen comparison is that a (non-linear) mean-variance relationship remains, despite the application of square root transformation as a variance stabilising option. Therefore, the choice of a horizontal variance cut-off is not appropriate when selecting highly variable genes. The same applies to Figures S2a, S3a and S3c. To account for such effect, a standard approach is to derive residual variance estimates after regressing out the mean-variance trend (see e.g. [7]). Highly variable genes can then be selected based on this residual measure of variability. Adopting this additional step is likely going to affect the choice of examples displayed in Figure 2c.

6.	Page 6. As per my previous comments, results obtained after selecting highly variable genes prior to dimensionality reduction may need to be updated.

7.	Figure S4. The authors may consider providing quantitative evidence (e.g. ARI after clustering) to complement the visual separation observed in the 2D t-SNE embedding (which may be affected by the choice of hyper-parameters).

Minor comments

1.	Page 3, line 40. Replace “see Figure 1h below” by “see Figure 1h”.

2.	Figure 1. Top panels present results associated to the PBMC dataset. Instead, most of the bottom panels correspond to synthetic data, yet panel f returns to the PBMC dataset. To avoid confusion, please provide an explicit description in the caption.

3.	Figure 1. Scatterplots include thousands for genes and therefore it is hard to visualise where are most genes concentrated. Please use a suitable colour-scheme, e.g. where dots are colour-coded according to their density.

References

[1]	Hafemeister and Satija (2019) https://doi.org/10.1186/s13059-019-1874-1

[2]	Townes et a! (2019) https://doi.org/10.1186/s13059-019-1861-6

[3]	Love et a! (2014) https://doi.org/10.1186/s13059-014-0550-8

[4]	Lun et al (2016) https://doi.org/10.1186/s13059-016-0947-7

[5]	Eling et a! (2018) https://doi.org/10.1016/j.cels.2018.06.011

[6]	Zappia et al (2017) https://doi.org/10.1186/s13059-017-1305-0

[7]	Amezquita et a! (2020) https://doi.org/10.1038/s41592-019-0654-x

**Reviewer 2**

**Are you able to assess all statistics in the manuscript, including the appropriateness of statistical tests used?**

Yes, and I have assessed the statistics in my report.

**Comments to author:**

Review of "Analytic Pearson residuals for normalization of single-cell RNA-seq UMI data" (GBIO-D-20-01910)

#summary

The authors presents a novel method for normalization of scRNA-seq UMI-based count data. The work aims at unifying two recently published approaches (sctransform by Hafemeister et al 2019 and GLM-PCA by Townes et al 2019), presenting a simplified method to Pearson residuals that can be used for downstream tasks in the analysis of scRNA-seq data (such as clustering, dimensionality reduction and visualization). The authors note that the GLM model with NB likelihood originally proposed in Hafemeister at al. is overspecified, thus showing inconsistent behaviour particularly for lowly-expressed genes (which motivated the regularization approach adopted by Hafemeister et al.). To overcome such limitation, the authors propose an "offset" model with a fixed slope and only one free parameter, for which there show an analytic solution, a desirable property with respect to computational costs. Furthermore, they show that the proposed "offset" model is equivalent to the rank-one GLM-PCA (from Townes et al. ), so essentially they discuss a special case of a published method.

Altogether, I find the generalization/summary interesting and potentially relevant, in particular learning about bias of existing methods etc. To convince a broad userbase, I'd like to see impact of this choice on bigger data, some downstream results etc. Please answer the simple question of impact for hands-on data analysts (maybe in connection to HVG, batch correction etc; see comments below, also maybe tie code into popular analysis frameworks such as Seurat or Scanpy).

#major comments

Can you please show impact of the choice of normalization method on one, two bigger examples - and a more detailed comparison of the results in S4/S5 going beyond visual comparison please.

The choice of fixing the overdispersion parameter theta to 100 is motivated empirically by estimating the parameter with negative control datasets. It would be interesting to see how the choice of such estimate (e.g. for theta=10,50,100) impacts downstream analysis results (e.g. ARI as per townes et al 2019 figure 5, qualitatively with tSNE etc.). This would give a good sense on the robustness of the approach.

Please use cluster metrics (such as ARI, NMI and others) to give a more quantitative evaluation of the method as compared to not only GLM-PCA and sctransform, but also other standard approaches such as normalization+median scaling etc. Similar to what the authors already did in Figure S6. This is also interesting because it's not so clear/strong how much all of these approaches (GLM-PCA, sctransform, offset model) really provide benefit compared to normalization+standard scaling. Since the latter is already the standard in common sc analysis pipelines, and very efficient, it would be interesting to run a more thorough evaluation of how much the impact of the normalization affects downstream analysis. This should also be done in large/complex dataset, the datasets chosed by the authors might not be sufficient (e.g. there should be a dataset with at least > 100k cells).

What is the % of HVG selected by sctransform vs. the offset model, what is the dependency to the mean expression? A critique made by Hafemesiter and Satja on the preprint version of the paper (https://satijalab.org/pdf/sctransform_offset.pdf) stated that several top genes ranked by variance with the offset model are not biologically relevant (mitochondrial, ribosomal). This is likely due to the constraint of the offset model to a fixed overdispersion value. It would not be concerning if the total subset of HV genes largely overlap, and thus cellular variation is preserved (although potentially with a slightly different feature set), but this needs to be shown explicitly. In a standard analysis workflow, the HVG selection is a crucial part, but the HVG scoring is not, therefore despite the larger weights given to mitochondrial/ribosomal genes by the offset model is not problematic per se, if a high % of biologically-relevant genes are maintained. But this needs to be shown clearly.

#minor comments

An interesting point that the authors note is that, for GLM-PCA, there seems to be missing within-cluster variation that leads to homogeneous clusters. This can also be seen e.g. in figure s5 for the normalization+sqrt transform. It would be interesting to quantitatively evaluate such an effect with a larger, more complex dataset.

a strength of the method is that it's computationally very efficient (if filtering is performed upfront as discussed). The paper only has one table (table S1) with such results. I would like to see a more thorough evaluation and report on multiple data sets.

**Authors Response**

**Point-by-point responses to the reviewers’ comments:**

Reviewer #1: Summary

Lause et al discuss some of the challenges arising in the normalisation of UMI-based single cell RNA sequencing (scRNAseq). The authors focus on specific issues arising from the model introduced by Hafemeister and Satija [1]. In particular, the authors argue that the model presented in [1] is over-specified and highlight challenges in the estimation of gene-specific over-dispersion parameters. Lause et al propose a simplification of the model in [1], which enables a close form solution for the associated Pearson residuals. This simplified model is shown to match a special case of the GLM-PCA method presented by Townes et al [2]. Finally, the authors compare the performance between the Pearson residuals mentioned above and an existing approach for normalisation and transformation of scRNAseq data prior to downstream analysis such as dimensionality reduction and clustering.

The authors raise an interesting discussion about the analysis of UMI-based scRNAseq data. However, I believe that some aspects of the analysis need to be improved before the article can be considered for publication. Moreover, some relevant literature is missing and direct links with other existing bulk and scRNAseq analysis tools is not provided (e.g. for the rank-one GLM-PCA model).Detailed comments are provided below.

*We thank the Reviewer for the overall positive assessment and excellent suggestions. See below for our point-to-point replies.*

Reviewer #1: Major comments

1.	Equation (3). This formulation matches what is known as global-scaling normalisation, where cell-specific scaling factors are used to capture overall differences across cells (e.g. sequencing depth or mRNA content). Such strategy is widely used in the context of bulk (e.g. [3]) and scRNAseq (e.g. [4]) analysis tools. As such, the authors should provide links to the relevant literature. Please note that whilst [1] and Lause et al use sequencing depth as cell-specific offsets ($n_c$), their use has been shown to be problematic and suitable alternatives (e.g. [4]) have been proposed.

*We thank the Reviewer for this comment. We did not mean to say that Equation (3) (now Equation (7), see also (2)) is novel. Of course the Reviewer is right and this is a quite common modeling assumption, as we also noted in the sentence introducing new Equation (2). The Reviewer is also right to suggest that additional citations can help to provide further context, so we inserted citations to [3] and [5] before new Equation (2) in the new introductory section 2.1, and also clarified that this is indeed a well-established framework.*

*Regarding size factors suggested in ref [4], we have inserted a reference to [4] but prefer to use sequencing depths because it leads to simple analytic expressions for Pearson residuals.*

2.	Page 3, line 25 (intercepts that showed zero variance). This statement is not accurate and needs to be clarified. Whilst the authors do observe that the intercept estimates are a deterministic function of the log-expression, this does imply that the associated estimator has “zero variance”. In fact, these estimates are subject to the same source of frequentist variance (e.g. as reflected by standard errors) as in maximum likelihood estimates. I suggest to rephrase as “intercept estimates were a deterministic function of log-expression (Figure 1e) as described in equation (4).

*Rephrased. This was indeed a sloppy formulation.*

3.	Equation (8). The proposed “rank-one GLM-PCA” approach matches existing count-based models used for bulk (e.g. [3]) and scRNAseq (e.g. [5]) data. In fact, the main difference relates to the strategy used to obtain parameter estimates (e.g. different choices for cell-specific scaling factors, regularisation of over-dispersion parameters). It is important to describe these links as the proposed model is not novel in itself, the main novelty relates to the explicit derivation of Pearson residuals (which is not done as a default option in the existing implementation of the existent models).

*We thank the Reviewer for this comment. Our comments above in response to issue 1 apply here. We inserted citations to [3] and [5] and clarified that this is a well-established framework.*

4.	Page 4. The authors argue that the procedure in [1] leads to biased over-dispersion estimates. This observation is supported by empirical results obtained from synthetic data and by highlighting convergence issues for the underlying estimation procedure.

a.	Maximum likelihood estimation for NB over-dispersion parameters is indeed problematic for lowly expressed genes and small sample sizes. This fact has been documented in the context of bulk [3] and scRNAseq [5] analysis, and suitable solutions have been proposed. Therefore, whilst the proposal of using a constant over-dispersion value across all genes may be a pragmatic solution to avoid the effect of this bias in the context of Pearson residuals, this is not necessarily required and better over-dispersion estimates could be used.

*We thank the reviewer for this comment and for bringing References [3] and [5] to our attention. The idea in those works is to model the relationship between the mean expression and the overdispersion estimate. This is conceptually similar to what Hafemeister & Satija do when they fit nonparametric regression to the overdispersion estimates (as in our Figure 1c). In fact, [5] used a similar nonparametric approach. We have inserted citations to [3] and [5] in this context (section 2.2).*

*However, our claim is that the initial overdispersion estimates that Hafemeister & Satija get are biased (as shown by our Figure 1f). The nonparametric regression will reduce the variance but not affect the bias: the estimated regression line is still biased. Therefore we do not think that any approaches based on post-hoc “smoothing” of the estimates can remedy the estimation bias.*

*More broadly, the Reviewer is correct that there may be better estimation procedures for the NB overdispersion parameter with less bias. In fact, we showed that simply increasing the number of iterations during inference already leads to less bias (Figure 1g). However, as we see no strong evidence for gene-specific over-dispersion parameters, we feel that a shared over-dispersion parameter is warranted for the reasons of parsimony. We clarified this point in the text (section 2.4).*

b.	The evidence provided using synthetic data and negative control datasets is not sufficient to support the use of a constant over-dispersion value across all genes. Whilst this may not have major consequences when deriving Pearson residuals, this does imply that a model with constant over-dispersion provides a better fit to real datasets in which underlying biological variability is present. To assess this, the strategy implemented by [6] could be used (the latter compares summary statistics derived from the raw data to those obtained from synthetic datasets generated by a range of competing models).

*Here we apologise for the misunderstanding. We did not mean to claim that constant over-dispersion provides a better fit to real datasets with biological variability! In fact, the opposite has to be true: datasets with biological variability will be better fit using gene-specific overdispersion, as this allows for differentially expressed genes to be modeled by a higher overdispersion compared to genes with constant expression across cells. However, instead of aiming at a perfect fit, our approach is different: For the purposes of defining Pearson residuals, we only want to account for technical variability; biological variability should then manifest itself as high residual variance. We edited the text to make this point clearer (section 2.5).*

c.	For the PBMC dataset, the reported estimation issues will primarily affect lowly expressed genes whose expression was only captured in a handful of cells. The current inclusion criteria applied by the authors is very liberal (16809 genes that were detected in at least five cells – as in [1]) and a more stringent filter will likely attenuate the issue.

*We agree with the reviewer. However, our Figure 1a-c simply replicates the analysis in Hafemeister and Satija (2019), and as they used 16809 genes, we did the same. In Figure 1f we further show that in the Hafemeister & Satija procedure, for the majority of the genes (yellow region) the overdispersion parameter estimates are affected by the estimation bias: genes with a mean expression below ~100 clearly diverge from the ground truth overdispersion. Note that some of the biologically variable genes selected by Hafemesiter & Satija have lower expression means (Figure 1h).*

*We discuss more stringent filters (such as selecting 1000 genes) later in the manuscript when we discuss HVG selection (section 2.6). This part of the paper was much extended in the revised manuscript, please see below.*

5.	Figure 2a. A critical limitation for the chosen comparison is that a (non-linear) mean-variance relationship remains, despite the application of square root transformation as a variance stabilising option. Therefore, the choice of a horizontal variance cut-off is not appropriate when selecting highly variable genes. The same applies to Figures S2a, S3a and S3c. To account for such effect, a standard approach is to derive residual variance estimates after regressing out the mean-variance trend (see e.g. [7]). Highly variable genes can then be selected based on this residual measure of variability. Adopting this additional step is likely going to affect the choice of examples displayed in Figure 2c.

*We absolutely agree with the reviewer. It is indeed true that many existing libraries (such as Seurat or Scanpy) perform HVG selection after regressing out the mean-variance relationship. This is usually done in some ad-hoc way (such as binning the average expression in 20 bins), often in a two-step procedure where mean-variance relationship is accounted for after log-transformation of the expression. We argue that Pearson residuals provide a more principled way to approach this problem and offer a simple and theoretically motivated criterion.*

*The second consideration behind our Figure 2a is that HVG selection is not the end of the preprocessing pipeline. The expression of selected genes is usually log- or sqrt-tranformed and then used for downstream tasks (such as PCA). These transformations are supposed to be variance-stabilizing but as we show in Figure 2a and Figure S3 (now S2, and much extended) both are unable to stabilize the variance across the entire range of gene expression.*

*We have clarified these points in the text surrounding Figure 2. We inserted a citation to [7].*

6.	Page 6. As per my previous comments, results obtained after selecting highly variable genes prior to dimensionality reduction may need to be updated.

*We have now performed a much more systematic comparison of different highly variable gene (HVG) criteria, including some that regress out the mean-variance relationship (Seurat, Seurat V3). See figure posted in the next issue.*

7.	Figure S4. The authors may consider providing quantitative evidence (e.g. ARI after clustering) to complement the visual separation observed in the 2D t-SNE embedding (which may be affected by the choice of hyper-parameters).

*We thank the reviewer for raising this issue.*

*In order to provide a quantitative comparison, we performed additional analysis on a dataset with known ground truth: a mixture of 8 FACS-sorted populations (Zheng et al., 2017, Duò et al., 2018), analyzed also in Townes et al. (2019) in their Figures 5 and S3. To make the dataset more challenging, we appended 10 pseudo-genes that had zero expression everywhere apart from 50 cells sampled from one of the populations; for these 50 cells these pseudo-genes had non-zero, but relatively low expression. This effectively created a ninth ground truth class, but with very small sample size (n=50), mimicking a rare but distinct cell type. We used 6 methods for highly variable gene (HVG) selection and 9 methods for data transformation and reduction to 50 dimensions, resulting in 6*9=54 combinations. We used kNN classification to assess the class separation in the 50-dimensional space, and used macro F1 score (harmonic mean of precision and recall, averaged across all classes) to quantify the performance as a single number. The results showed that Pearson residuals HVG followed by Pearson residual transformation performed the best in isolating ground truth clusters. For a detailed discussion, see paper.*

Reviewer #1: Minor comments

1.	Page 3, line 40. Replace “see Figure 1h below” by “see Figure 1h”.

*Fixed.*

2.	Figure 1. Top panels present results associated to the PBMC dataset. Instead, most of the bottom panels correspond to synthetic data, yet panel f returns to the PBMC dataset. To avoid confusion, please provide an explicit description in the caption.

*We edited the caption to avoid confusion, and also added titles to the Figure panels that show simulated data. The updated figure is shown below.*

3.	Figure 1. Scatterplots include thousands for genes and therefore it is hard to visualise where are most genes concentrated. Please use a suitable colour-scheme, e.g. where dots are colour-coded according to their density.

*We colored points by local density in all panels apart from panel H (where color is already used to highlight selected genes).*

References

[1]	Hafemeister and Satija (2019) https://doi.org/10.1186/s13059-019-1874-1

[2]	Townes et a! (2019) https://doi.org/10.1186/s13059-019-1861-6

[3]	Love et a! (2014) https://doi.org/10.1186/s13059-014-0550-8

[4]	Lun et a! (2016) https://doi.org/10.1186/s13059-016-0947-7

[5]	E!ing et a! (2018) https://doi.org/10.1016/j.ce!s.2018.06.011

[6]	Zappia et a! (2017) https://doi.org/10.1186/s13059-017-1305-0

[7]	Amezquita et a! (2020) https://doi.org/10.1038/s41592-019-0654-x

Reviewer #2 Summary

The authors presents a novel method for normalization of scRNA-seq UMI-based count data. The work aims at unifying two recently published approaches (sctransform by Hafemeister et al 2019 and GLM-PCA by Townes et al 2019), presenting a simplified method to Pearson residuals that can be used for downstream tasks in the analysis of scRNA-seq data (such as clustering, dimensionality reduction and visualization). The authors note that the GLM model with NB likelihood originally proposed in Hafemeister at al. is overspecified, thus showing inconsistent behaviour particularly for lowly-expressed genes (which motivated the regularization approach adopted by Hafemeister et al.). To overcome such limitation, the authors propose an "offset" model with a fixed slope and only one free parameter, for which there show an analytic solution, a desirable property with respect to computational costs. Furthermore, they show that the proposed "offset" model is equivalent to the rank-one GLM-PCA (from Townes et al. ), so essentially they discuss a special case of a published method.

Altogether, I find the generalization/summary interesting and potentially relevant, in particular learning about bias of existing methods etc. To convince a broad userbase, I'd like to see impact of this choice on bigger data, some downstream results etc. Please answer the simple question of impact for hands-on data analysts (maybe in connection to HVG, batch correction etc; see comments below, also maybe tie code into popular analysis frameworks such as Seurat or Scanpy).

*We thank the reviewer for the encouraging assessment. Please see below for point-to-point to replies.*

*Regarding the code, we are working on integrating our pipeline into Scanpy: https://github.com/theislab/scanpy/pull/1715. The Scanpy team expressed interest in including this into the core Scanpy functionality. We are going to work on merging this pull request while the manuscript is in the second round of review. Additionally, we are updating our own Github repository to use core Scanpy functions wherever possible.*

Reviewer #2: Major comments

1. Can you please show impact of the choice of normalization method on one, two bigger examples - and a more detailed comparison of the results in S4/S5 going beyond visual comparison please.

*We thank the reviewer for these suggestions.*

*First, we performed additional analysis on the mouse developmental dataset with 2 million cells from Cao et al. (2019). We show that analytic Pearson residuals yield good results when used for gene selection and also for data transformation. We also show that it works very fast (4 min) whereas e.g. GLM-PCA required 52 hours to converge.*

*When analyzing this dataset with the suggested Pearson residual pipeline, we unexpectedly discovered that several genes were highly expressed in small subsets of cells, with near-zero expression everywhere else, and those subsets of cells were always all from one of the embryos. These subsets were clearly visible as isolated islands on a t-SNE plot. After we filtered out the batch-effect-driven genes, the resulting t-SNE did not show this artifact anymore. Notably, Pearson residuals was the only method that uncovered this peculiar batch effect. We consider it a demonstration of the strength and sensitivity of the method: the genes that had artifact expression in one of the embryos were (correctly) selected as ‘variable’ and led to the appearance of isolated populations expressing those genes.*

*Second, in order to provide a quantitative comparison, we performed additional analysis on a dataset with known ground truth: a mixture of 8 FACS-sorted populations (Zheng et al., 2017, Duò et al., 2018), analyzed also in Townes et al. (2019) in their Figures 5 and S3. To make the dataset more challenging, we appended 10 pseudo-genes that had zero expression everywhere apart from 50 cells sampled from one of the populations; for these 50 cells these pseudo-genes had non-zero, but relatively low expression. This effectively created a ninth ground truth class, but with very small sample size (n=50), mimicking a rare but distinct cell type. We used 6 methods for highly variable gene (HVG) selection and 9 methods for data transformation and reduction to 50 dimensions, resulting in 6*9=54 combinations. We used kNN classification to assess the class separation in the 50-dimensional space, and used macro F1 score (harmonic mean of precision and recall, averaged across all classes) to quantify the performance as a single number. The results showed that Pearson residuals HVG followed by Pearson residual transformation performed the best in isolating ground truth clusters. For a detailed discussion, see paper.*

2.	The choice of fixing the overdispersion parameter theta to 100 is motivated empirically by estimating the parameter with negative control datasets. It would be interesting to see how the choice of such estimate (e.g. for theta=10,50,100) impacts downstream analysis results (e.g. ARI as per townes et al 2019 figure 5, qualitatively with tSNE etc.). This would give a good sense on the robustness of the approach.

*We have now included theta=10 (in addition to our default choice of theta=100) into the comparison using the ground truth dataset (see above). The results were very similar.*

3.	Please use cluster metrics (such as ARI, NMI and others) to give a more quantitative evaluation of the method as compared to not only GLM-PCA and sctransform, but also other standard approaches such as normalization+median scaling etc. Similar to what the authors already did in Figure S6. This is also interesting because it's not so clear/strong how much all of these approaches (GLM-PCA, sctransform, offset model) really provide benefit compared to normalization+standard scaling. Since the latter is already the standard in common sc analysis pipelines, and very efficient, it would be interesting to run a more thorough evaluation of how much the impact of the normalization affects downstream analysis. This should also be done in large/complex dataset, the datasets chosed by the authors might not be sufficient (e.g. there should be a dataset with at least > 100k cells).

*We thank the reviewer for this suggestion. Please see the figure directly above (Figure 5 in the revised manuscript) for a thorough comparison of 54 different pipelines on a dataset with known ground truth.*

*For the quantitative benchmark comparison we decided to use kNN classification accuracy to assess the class separation (see above; the class labels were separate FACS-sorting experiments). We find this a more objective and transparent approach compared to ARI/NMI/etc because the latter metrics would require us to cluster the data and compare the clustering results with the known classes. The end result could strongly depend on the clustering method of choice, so we decided to use a classification approach to avoid these complications. Also, kNN classification accuracy usually corresponds well to the visual impression of the t-SNE visualizations (this makes sense as t-SNE/UMAP/etc aim to preserve nearest neighbors).*

*We are not aware of any larger dataset with known ground truth. We are reluctant to use datasets without known ground truth for quantitative benchmarking because this would require treating inferred cell type labels (usually derived via clustering) as ground truth, which may strongly depend on whatever procedure was used by original authors to do the original clustering and hence may bias the benchmarking comparison. For qualitative assessment, we added the new 2-million-cell dataset to show that the method also works for large datasets.*

4. What is the % of HVG selected by sctransform vs. the offset model, what is the dependency to the mean expression? A critique made by Hafemesiter and Satja on the preprint version of the paper (https://satijalab.org/pdf/sctransform_offset.pdf) stated that several top genes ranked by variance with the offset model are not biologically relevant (mitochondrial, ribosomal). This is likely due to the constraint of the offset model to a fixed overdispersion value. It would not be concerning if the total subset of HV genes largely overlap, and thus cellular variation is preserved (although potentially with a slightly different feature set), but this needs to be shown explicitly. In a standard analysis workflow, the HVG selection is a crucial part, but the HVG scoring is not, therefore despite the larger weights given to mitochondrial/ribosomal genes by the offset model is not problematic per se, if a high % of biologically-relevant genes are maintained. But this needs to be shown clearly.

*We thank the reviewer for this suggestion. We have now included an explicit figure (Figure S2 in the revised manuscript) comparing the 100 HVG selection by scTransform with default parameters and the 100 HVG selection by analytic Pearson residuals using the PBMC dataset analysed in the original Hafemeister & Satija paper. The selected sets are almost identical (87 genes in common; overlap between 1000 HVG sets is 834), as shown in the following figure (S2), panel E:*

*In their critical response, H&S listed 10 genes with Pearson residual variance exceeding the scTransform variance, sorted by the magnitude of the difference (note that they used a different version of the PMBC dataset in their response). In the PMBC dataset we analyzed here, the top 10 genes by this criterion are as follows: Malat1, B2m, Tmsb4x, Actb, Rps6, Rpl13, Rps27a, Rpl13a, Rpl3, Rps3a. There are indeed many ribosomal genes in this list. But most of these actually have relatively small variances in the Pearson residual representation. So we believe that the agreement to scTransform is in fact very good (compare panels A and E above). Also, note that we explicitly argue in the text that Malat1, included in the above list, shows biologically meaningful variation, despite scTransform not selecting it among 100 HVGs (it comes out 224th in scTransform but 20th with analytic Pearson residuals with theta=100).*

*This is not directly related to this issue, but note that the above figure (S2 in the manuscript) also shows variance of the deviance residuals, which were suggested by Townes et al. as a “fast approximation” to GLM-PCA. Our conclusion is that deviance residuals are not suitable either for HVG selection or for downstream tasks because they do not stabilize the variance across the expression means, unlike Pearson residuals.*

*The above figure (S2) is an extension of what used to be Figure S3 in the original submission; it contained panels A, B, and C, but without log-scaling the vertical axis. We now prefer this log-scaled version as it better highlights the trends in the data. We switched the corresponding panels in Figure 2 to log-scale as well:*

*And also for former Figure S2 (now Figure S3) (different dataset):*

*Reviewer #2: Minor comments*

*1.	An interesting point that the authors note is that, for GLM-PCA, there seems to be missing within-cluster variation that leads to homogeneous clusters. This can also be seen e.g. in figure s5 for the normalization+sqrt transform. It would be interesting to quantitatively evaluate such an effect with a larger, more complex dataset.*

*We think this effect may be related to problems GLM-PCA has with convergence. It is indeed very clearly visible using the n=2mln dataset, as shown above. For quantitative comparison, see the ground-truth dataset (shown above) where we also see that GLM-PCA performs worse.*

*2.	a strength of the method is that it's computationally very efficient (if filtering is performed upfront as discussed). The paper only has one table (table S1) with such results. I would like to see a more thorough evaluation and report on multiple data sets.*

*We have now moved that table to the main text (now as Table 1) and added the n=2mln dataset in there. Here is the resulting table:*

**Second round of review**

**Reviewer 1**

Referee report for GBIO-D-20-01910-R1

Analytic Pearson residuals for normalization of single-cell RNA-seq UMI data

The authors have addressed most of the comments that I raised after their first submission. I believe that manuscript and its structure has substantially improved. However, some of my concerns still remain and I suggest the authors to revise the document accordingly.

Major comments

*Author’s response to comment 4a*

*We thank the reviewer for this comment and for bringing References [3] and [5] to our attention. The idea in those works is to model the relationship between the mean expression and the overdispersion estimate. This is conceptually similar to what Hafemeister & Satija do when they fit nonparametric regression to the overdispersion estimates (as in our Figure 1c). In fact, [5] used a similar nonparametric approach. We have inserted citations to [3] and [5] in this context (section 2.2).*

*However, our claim is that the initial overdispersion estimates that Hafemeister & Satija get are biased (as shown by our Figure 1f). The nonparametric regression will reduce the variance but not affect the bias: the estimated regression line is still biased. Therefore we do not think that any approaches based on post-hoc “smoothing” of the estimates can remedy the estimation bias.*

*More broadly, the Reviewer is correct that there may be better estimation procedures for the NB overdispersion parameter with less bias. In fact, we showed that simply increasing the number of iterations during inference already leads to less bias (Figure 1g). However, as we see no strong evidence for gene-specific over-dispersion parameters, we feel that a shared over-dispersion parameter is warranted for the reasons of parsimony. We clarified this point in the text (section 2.4).*

Further comments:

Indeed, the approaches in [3] and [5] are conceptually similar to the correction proposed by Hafemeister & Satija. However, there is an important difference. Whilst Hafemeister & Satija introduce the shrinkage in a post-hoc manner, both [3] and [5] incorporate the shrinkage in a unified estimation procedure (empirical Bayes and a fully Bayesian approach, respectively). As such, [3] and [5] can mitigate estimation bias for over-dispersion parameters. To clarify this, I suggest to edit the last paragraph in page 2 as follows. Replace: “(similar to Love et al, 2014; Eling et al, 2018).” by “This is similar to Love et al (2014) and Eling et al (2018), but uses a post-hoc correction instead of incorporating shrinkage in a unified estimation procedure”.

*Author’s response to comment 4b*

*Here we apologise for the misunderstanding. We did not mean to claim that constant over-dispersion provides a better fit to real datasets with biological variability! In fact, the opposite has to be true: datasets with biological variability will be better fit using gene-specific overdispersion, as this allows for differentially expressed genes to be modeled by a higher overdispersion compared to genes with constant expression across cells. However, instead of aiming at a perfect fit, our approach is different: For the purposes of defining Pearson residuals, we only want to account for technical variability; biological variability should then manifest itself as high residual variance. We edited the text to make this point clearer (section 2.5).*

Further comments:

Many thanks for the clarification. I agree, constant over-dispersion may be sufficient in the absence of biological variability (i.e. negative control datasets) but not otherwise. Whilst the authors have edited some of the text to clarify this, some of the remaining statements can be misleading. For example, the abstract states “the data are in fact consistent with constant over-dispersion parameter across genes”. Similarly, the introduction section says “UMI data do not require gene-specific overdispersion parameters”. Such statements need to be edited to provide the same clarification described above: they are only true in the absence of biological variability and constant overdispersion only aims to capture technical variability and biological variability is expected to be reflected as high residual variance.

*Author’s response to comment 4c*

*We agree with the reviewer. However, our Figure 1a-c simply replicates the analysis in Hafemeister and Satija (2019), and as they used 16809 genes, we did the same. In Figure 1f we further show that in the Hafemeister & Satija procedure, for the majority of the genes (yellow region) the overdispersion parameter estimates are affected by the estimation bias: genes with a mean expression below ~100 clearly diverge from the ground truth overdispersion. Note that some of the biologically variable genes selected by Hafemesiter & Satija have lower expression means (Figure 1h).*

*We discuss more stringent filters (such as selecting 1000 genes) later in the manuscript when we discuss HVG selection (section 2.6). This part of the paper was much extended in the revised manuscript, please see below.*

Further comments:

I agree, using the same filter as In Hafemeister and Satija is important to provide a direct comparison. As the issue of bias is primarily discussed in Section 2.4, I suggest to mention this issue there. For example, the authors could add the following sentence at the end of their 3rd paragraph: “These numerical issues diminish when more stringent gene filtering criteria are used as part of quality control steps”.

*Author’s response to comment 5*

*We absolutely agree with the reviewer. It is indeed true that many existing libraries (such as Seurat or Scanpy) perform HVG selection after regressing out the mean-variance relationship. This is usually done in some ad-hoc way (such as binning the average expression in 20 bins), often in a two-step procedure where mean-variance relationship is accounted for after log-transformation of the expression. We argue that Pearson residuals provide a more principled way to approach this problem and offer a simple and theoretically motivated criterion.*

*The second consideration behind our Figure 2a is that HVG selection is not the end of the preprocessing pipeline. The expression of selected genes is usually log- or sqrt-tranformed and then used for downstream tasks (such as PCA). These transformations are supposed to be variance-stabilizing but as we show in Figure 2a and Figure S3 (now S2, and much extended) both are unable to stabilize the variance across the entire range of gene expression.*

*We have clarified these points in the text surrounding Figure 2. We inserted a citation to [7].*

Further comments:

As Figure 2 still directly compares panels a and b, I suggest to add the following text to the 4th paragraph in Section 2.6. “... square-root transform is not sufficient for variance stabilization and further modelling would be required to remove the mean-variance confounding”.

**Reviewer 2**

Review of “Analytic Pearson residuals for normalization of single-cell RNA-seq UMI data” (GBIO-D-20-01910)

Summary

The authors presented a revised version of the article that largely addresses the previous comments, congratulations for that!

The performance on larger datasets as well as the running time comparisons are convincing.

It's interesting how the analysis on a large, more complex dataset highlighted an additional "batch effect" across samples. It is unclear however from the figure, and only briefly described in the text and caption. It would be useful to interpret and visualize better such results: what was the proportion of samples affected? Which samples and were they always the same? Furthermore, the heuristic chosen for the filtering seems a bit ad hoc and would be interesting to understand when such filtering breaks. The analysis also highlighted how standard CPMedian seems to be robust for such cases.

The classifier performance on the ground truth dataset is also convincing and highlights how both feature selection and normalization has an impact to discover true clusters. The Pearson residual selection also seems to improve consistently across data transformation strategies.

The comparison of feature selection with pearson residuals and sctransform is also more extensive and it addresses my previous comment. The interpretation on the genes for which the methods differ is also clear.

**Authors Response**

**Point-by-point responses to the reviewers’ comments:**

Reviewer 1:

The authors have addressed most of the comments that I raised after their first submission. I believe that manuscript and its structure has substantially improved. However, some of my concerns still remain and I suggest the authors to revise the document accordingly.

*We would again like to thank the reviewer for very useful feedback!*

*Please note that in addition to addressing the reviewers’ comments, we extended the benchmark shown in Figure 5, and added some further points and references into the Results and the Discussion. These relate to the similarity to the correspondence analysis (in the Results); to the parallel paper suggesting a method called Sanity (Discussion); and to the follow-up papers from the Satija lab and the Huber lab (Discussion). Please see the tracked changes version of the manuscript.*

Indeed, the approaches in [3] and [5] are conceptually similar to the correction proposed by Hafemeister & Satija. However, there is an important difference. Whilst Hafemeister & Satija introduce the shrinkage in a post-hoc manner, both [3] and [5] incorporate the shrinkage in a unified estimation procedure (empirical Bayes and a fully Bayesian approach, respectively). As such, [3] and [5] can mitigate estimation bias for over-dispersion parameters. To clarify this, I suggest to edit the last paragraph in page 2 as follows. Replace: “(similar to Love et al, 2014; Eling et al, 2018).” by “This is similar to Love et al (2014) and Eling et al (2018), but uses a post-hoc correction instead of incorporating shrinkage in a unified estimation procedure”.

*We agree, and have edited accordingly.*

Many thanks for the clarification. I agree, constant over-dispersion may be sufficient in the absence of biological variability (i.e. negative control datasets) but not otherwise. Whilst the authors have edited some of the text to clarify this, some of the remaining statements can be misleading. For example, the abstract states “the data are in fact consistent with constant over-dispersion parameter across genes”. Similarly, the introduction section says “UMI data do not require gene-specific overdispersion parameters”. Such statements need to be edited to provide the same clarification described above: they are only true in the absence of biological variability and constant overdispersion only aims to capture technical variability and biological variability is expected to be reflected as high residual variance.

*This is a good point, and we have edited these sentences accordingly. Please see the exact edits in the file with tracked changes.*

I agree, using the same filter as In Hafemeister and Satija is important to provide a direct comparison. As the issue of bias is primarily discussed in Section 2.4, I suggest to mention this issue there. For example, the authors could add the following sentence at the end of their 3rd paragraph: “These numerical issues diminish when more stringent gene filtering criteria are used as part of quality control steps”.

*We have inserted a remark along these lines.*

As Figure 2 still directly compares panels a and b, I suggest to add the following text to the 4th paragraph in Section 2.6. “... square-root transform is not sufficient for variance stabilization and further modelling would be required to remove the mean-variance confounding”.

*We are now ending the Section 2.6 with a comment on modeling the mean-variance relationship (which serves as a lead to the next section), including a similar statement.*

Reviewer #2

The authors presented a revised version of the article that largely addresses the previous comments, congratulations for that!

*We would again like to thank the reviewer for very useful feedback!*

*Please note that in addition to addressing the reviewers’ comments, we extended the benchmark shown in Figure 5, and added some further points and references into the Results and the Discussion. These relate to the similarity to the correspondence analysis (in the Results); to the parallel paper suggesting method called Sanity (Discussion); and to the follow-up papers from the Satija lab and the Huber lab (Discussion). Please see the tracked changes version of the manuscript.*

The performance on larger datasets as well as the running time comparisons are convincing.

It's interesting how the analysis on a large, more complex dataset highlighted an additional "batch effect" across samples. It is unclear however from the figure, and only briefly described in the text and caption. It would be useful to interpret and visualize better such results: what was the proportion of samples affected? Which samples and were they always the same? Furthermore, the heuristic chosen for the filtering seems a bit ad hoc and would be interesting to understand when such filtering breaks. The analysis also highlighted how standard CPMedian seems to be robust for such cases.

*We agree that this batch effect is very interesting, but we believe deeper exploration would be beyond the scope of our manuscript. We have discussed this issue with the authors of the original study, and they suggested it may be due to some batch-specific confusion between embryo barcode and a particular transcript location. We have now extended the caption of our supplementary figure S6 to say that “Almost every embryo exhibited some of these spuriously enriched genes.”*

*For the interested reader, we provide the following plots in our github-repository (notebook 091_caodata.ipynb).*

*This first plot illustrates that almost all embryos are affected (blue dots), by showing how many of the excluded genes were strongest in which embryo.*

*This second plot shows that, for excluded genes (top row), typically only one or very few embryos showed strong expression of that gene (only one or few squares per column). In contrast, among non-excluded genes (bottom row), embryos showed similar strength for the same gene. Note that “white” codes for 0 cells with “strong” expression here.*

The classifier performance on the ground truth dataset is also convincing and highlights how both feature selection and normalization has an impact to discover true clusters. The Pearson residual selection also seems to improve consistently across data transformation strategies. The comparison of feature selection with pearson residuals and sctransform is also more extensive and it addresses my previous comment. The interpretation on the genes for which the methods differ is also clear.

*We thank the reviewer for these comments.*
